# Cytoskeletal Tensegrity in Microgravity

**DOI:** 10.3390/life11101091

**Published:** 2021-10-15

**Authors:** John Gardiner

**Affiliations:** Independent Researcher, Glebe 2037, Australia; jgardiner88@bigpond.com

**Keywords:** actin, cytoskeleton, microgravity, microtubule, tensegrity

## Abstract

In order for Man to venture further into Space he will have to adapt to its conditions, including microgravity. Life as we know it has evolved on Earth with a substantial gravitational field. If they spend considerable time away from Earth, astronauts experience physiological, mental, and anatomical changes. It is not clear if these are pathological or adaptations. However, it is true that they experience difficulties on their return to stronger gravity. The cytoskeleton is a key site for the detection of gravitational force within the body, due to its tensegrity architecture. In order to understand what happens to living beings in space, we will need to unravel the role cytoskeletal tensegrity architecture plays in the building and function of cells, organs, the body, and mind.

“If we’re going to go to the moon again and we’re going to go to Mars and beyond, then we’ve got to get a little outside of our comfort zone” (Jared Isaacman in *The Guardian* 16th September 2021).

## 1. Definition

**Figure 1:**
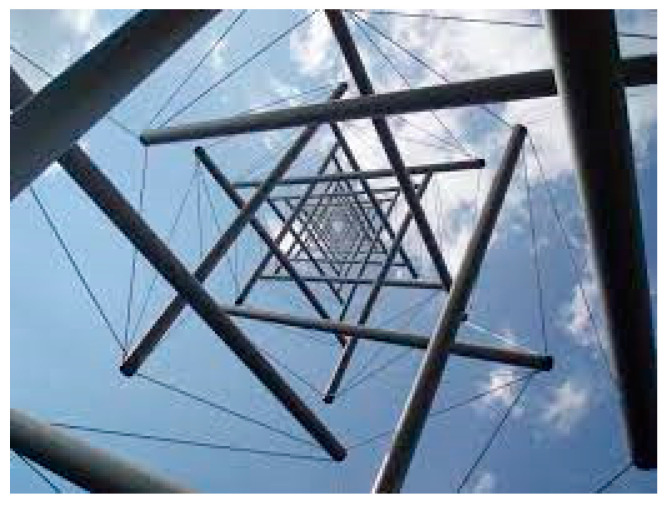
“Needle Tower” by Kenneth Snelson demonstrating tensegrity architecture with compression-resistant and tensional elements.

“Tensegrity is a design principle that applies when a discontinuous set of compression elements is opposed and balanced by a continuous tensile force, thereby creating an internal prestress that stabilizes the entire structure.” [1] See Figure 1.

## 2. Tensegrity and the Universe including Life

Buckminster Fuller, the mathematician, first used the term “tensegrity” [2]. It should be noted that his student, the renowned artist Kenneth Snelson, preferred the term “floating compression”. Fuller thought that stars’ and planets’ compression elements were held in place by prestress tensional gravity. He also saw this—*discontinuous compression, continuous tension*—in atoms, electrons orbiting the nucleus and it all held together by subatomic attraction and repulsion. Thus, he saw that the Universe is a tensegrity fractal, with self-similarity at different scales. This enables a hierarchical nesting of living beings’ structure. Thus, the gravitational force acts at the whole organ or tissue level and is sensed at cellular and molecular levels. Indeed, gravity can lead to changes in many cellular functions, including proliferation, differentiation, signalling, and expression. Conversely, changes at the subatomic and molecular level can be translated into more macro-occurrences [3,4]. Just as gravity “prunes” all that is not part of a constructed building, biotensegrity “prunes” all that is not part of the great fractal continuum from micro to macro and vice versa, thus, acting as an ultimate proof-reading algorithm [5].

## 3. Tensegrity and the Body

Tensegrity lends itself to describing biomechanical joints like the elbow, whichare stable during shape changes due to “prestress”. Connective tissues integrate components into a single unit [6]. Indeed, the entire human frame with its many tensile components pulling on rigid bones and stabilising them against gravity, is tensegrity [1].

Furthermore, even human tensegrity architecture is part of this continuum. Richard Dawkins [7] makes the point that the phenotype of an organism is not limited to the actual body of that organism but extends to the ways in which the organism modifies its surrounding environment. Thus, a bird’s nest is the extended phenotype of a bird. A beaver’s dam is the extended phenotype of a beaver, and tensegrity-based architecture is the extended phenotype of humans. So, the human mind’s functioning is somehow based in tensegrity. “Embodiment” in robotics means that the robot body and its controller are not considered separate but that the interaction of the body with its environment is taken into account. This applies to tensegrity structures. Thus, the human brain may compute without need to invoke any special “soul” or atman because it is a tensegrity structure [8]. Interestingly, the nervous system can be seen as fractal, with hierarchical structure progressing from the micro to the macro and vice versa [9]. The brain is a tensegrity structure of its grey and white matters and fascia. These in turn are constructed of tensegrity assemblages of cells, then actin and MT cytoskeletons and attractive (tensional) and repulsive (compressive) forces between individual amino acids and so forth.

“The conception of a haptic medium as a nested tensegrity structure has been proposed to express the obtained information realized by myofascia deformation, by its invariants and transformations. The tensegrity proposal rationalizes the relative indifference of dynamic touch to the site of mechanical contact (hand, foot, torso or probe) and the overtness of exploratory activity. It also provides a framework for dynamic touching’s fractal nature, and the finding that its degree of fractality may matter to its accomplishments” [10]. Indeed.

## 4. Qualities of Tensegrity Structures: The Cytoskeleton

Tensegrity structures with discontinuous members are used both in terrestrial, outer-space, and morphing devices [11]. Composed of discontinuous struts and cables, these systems are only structurally stable when prestress is induced; otherwise, they lose their geometrical configuration (while keeping its topology) and, thus, can be tightly packed. This enables gigantic shape change, making such structures highly versatile and resilient [11]. These qualities also make nested hierarchies of tensegrity ideal for biological systems to create relatively large structures with a minimum of protein subunits. This includes cytoskeletal tensegrities.

Due to internal prestress in the cytoskeleton, cells take on a spherical shape when in suspension. However, if a cell is anchored to a resisting substrate, it spreads out taking on a flattened form. When cells are attached to a flexible substrate, it is pulled into compression wrinkles [12]. Both cell–cell and cell–extracellular matrix (ECM) adhesions have unique components, but both are linked to the cell’s actin cytoskeleton. Thus, mechanical and/or chemical stimuli are transferred and integrated to induce or control cell proliferation, migration, and differentiation [13]. Focal adhesions have hundreds of clustered integrins and other cytoplasmic proteins connecting with hundreds of actin and myosin (motor protein) proteins. This enables the force at a single focal adhesion to be tens of nN and the force can propagate along the cytoskeleton distances up to tens of µm. The size of a cell. The cytoskeleton mediating long distance force transmission in the cytoplasm has been shown in several cell types [14].

Actin is involved in mechanosensing through other pathways. In a process known as cytoskeletal strengthening, integrin-mediated cell–ECM adhesion is enhanced through interactions with cytoskeletal proteins, among which vinculin is important in stimulating actin polymerisation and recruiting actin remodelling proteins [15]. Cadherins are a large superfamily of calcium-dependent transmembrane adhesive proteins that are responsible for cell–cell adhesion in all soft tissues [16]. Extracellular regions of cadherin mediate the adhesion between neighbouring cells, while the cytoplasmic tails of cadherins interact with effector proteins, which connect them to the actin cytoskeleton [17].

The microtubule (MT) cytoskeleton can transmit mechanical signals and resist compression, thus, fulfilling its role as the compression-resistant component of subcellular cytoskeletal tensegrity. It is, along with the actin cytoskeleton, central to the perception of external forces by cells [18]. In contracting cardiomyocytes detyrosination of MTs (a post-translational modification of MTs) promoted buckling, stiffened the myocytes, and correlated with impaired function in cardiomyopathy. Thus, detyrosinated MTs represent crucial compression-resistant cytoskeletal components [19]. Importantly, MT post-translational modifications affect the binding of partner proteins, such as the motor proteins kinesins to the MT. MTs adhered to a two-dimensional substrate by MT-associated protein kinesin buckle when subjected to compression stress. The density of kinesin is found to play the key role in determining the buckling mode of MTs [20]. In fact, the cardiac MT network may act as a cytoskeletal shock-absorber [21].

## 5. Tensegrity and the Nervous System

The cytoskeletons of the brain and nervous systems may function as tensegrity systems. Nerve axons typically possess bundled MTs and the dendritic spines polymerised actin filaments. If MTs in the nerve-axon are in compression as in tensegrity, then pulling on the axon could reduce the compression. Normally, this is done by a growth cone but this could also be done by pulling. In either case, a decrease in the compression of the assembled MTs should favour MT assembly, and this is what is observed [22]. Various other models of the brain suggest the brain and nervous systems as tensegrity structures [23]. A fascial layer (prestressed structure) covers brain, spinal cord, and many nerves. The fascia mechanically connects neural tissue (compression-resistant structures) to the rest of the body [24]. Indeed, the brain even pulses, with the spinal cord acting as a piston [25], the force possibly dissipated through the brain’s cytoskeletal tensegrity network as heat.

## 6. Graviception and the Cytoskeleton

Cells probably sense changes in gravitational force through alterations in forces between their adhesions and the cytoskeleton or through force acting directly on the cytoskeleton, rather than through a “gravioreceptor” molecule. The entire cell or even entire tissue is a gravity sensor [26]. Local bending of the cytoskeleton is common to all cellular mechanotransduction. Mechanosensory cells experience gravitational force through dense organelles (e.g., otoliths, stereocilia). Other cells also feel gravitational force as cytoskeletal distortion, perhaps compression resistant elements (MT) tugging on prestressed tensional elements (actin filaments). Indeed, in human primary endothelial cells, there are alterations to the actin cytoskeleton under both hypogravity and hypergravity [27]. Another study found a collapse of the actin cytoskeleton of osteoblasts after four days of microgravity [28].

When organisms experience microgravity, they experience an acute decrease in pre-stress on the macro (tissue, organ) level, which, due to the hierarchical organisation of life, trickles down to produce corresponding changes in form and function at the cellular and molecular levels including an upregulation of cytoskeletal genes in cancer cells [29]. Cells derived from the nervous systems are affected. Under simulated microgravity, the adhesion of primary cells from human brain nervous tissue was decreased, accompanied by highly disorganized β-tubulin structures in a circular pattern around the nucleus [30]. In human SH-SY5Y neuroblastoma cells, weightlessness or changing gravity resulted in microtubular bending and loop formation [31].

The third class of cytoskeletal proteins, intermediate filaments, is also involved in the tensegrity architecture of human cells forming tensile cables that link the cell nucleus to its cortex [32]. There is evidence that intermediate filaments play a role in cells’ response to gravity (or lack thereof) [33].

## 7. Changes to Tissues in Microgravity

Simulated microgravity alters morphology, cytoskeletal organisation, and cellular motility in primary neurons as well as cytoskeleton protein expression in rat hypothalamus and mouse hippocampus and hypothalamus [34]. Neurons are in functionally involved in human adaptation to microgravity. After short exposures to simulated microgravity, neurons changed morphology followed by fast recovery on return to Earth surface gravity. Long exposure to microgravity resulted in possible adaptation of single neurons as well as neuronal networks. The latter was coupled to an increase of apoptosis. However, neurons and neuronal networks exposed for long-term to microgravity required longer recovery time to re-adapt to Earth surface gravity [35]. One possible irreversible change to the nervous system under microgravity is the hyperbranching of motor neurons [36].

Another change seen under microgravity is muscular atrophy. As mentioned above, muscles act as tensional elements in the musculoskeletal body. Thus, microgravity leads to the breakdown of this hierarchy of the body fractal. The muscle consists in a large part of actin filaments, which act in conjunction with myosin to provide the force of muscle contraction. Under microgravity, the expression of actin and myosin are altered [37] so changes in one iteration of the body fractal (cytoskeletal level) appear to have consequences at higher hierarchies. Bone is affected. Primary cilia act as microgravity sensors, depolymerising microtubules to inhibit osteolast differentiation and mineralisation [38].

Interestingly, one study found neither quantitative nor structural changes in the actin cytoskeleton of macrophages under microgravity [39]. The cells of the immune system are not directly involved in the cytoskeleton/tissue/organ hierarchies of fractal hierarchy. Thus, they may be spared the potentially negative effects that microgravity has upon these tensegrity-based systems.

## 8. Microgravity May Promote Mental Illness

Three of the most prevalent mental illnesses are depression, bipolar syndrome, and schizophrenia. Mental illnesses are not quite the same as neurological disorders, although there is certainly some overlap. Whereas with many neurological diseases it is possible to say THIS disorder is caused by THIS mutation and THIS process; in general mental (psychiatric) illnesses have a much more nebulous aetiology. Of course, there are exceptions, and I am sure many neurologists and psychiatrists would disagree! Machado-Joseph disease, despite its prevalence remains unsolved [40]; a gene for schizophrenia in a Scottish lineage, *DISC1*, has been identified [41].

In a previous paper [42], I suggested that depression, bipolar syndrome, and schizophrenia (and other conditions besides) may be malfunctions of the nervous system cytoskeletal tensegrity framework, particularly in the brain. Indeed, a tensegral understanding is beginning to be developed for treatments in allied health including osteopathy [43] and in oncological diagnosis [44]. In depression, the nervous system cytoskeleton may be too “floppy”, there might not be enough prestress, and in schizophrenia too “taut” with too much prestress. Bipolar syndrome is a condition that fluctuates between these extremes. There is certainly abundant evidence that the cytoskeleton plays a key role in mental health [45,46].

A number of psychiatric and psychological hurdles have arisen for astronauts [47]. In particular, there seems to be a tendency for them to develop anxiety- and depression-spectrum disorders [48]. These have typically been seen as psychological manifestations of loneliness, isolation, interpersonal stress. However, as I have laid out above, microgravity’s effect on the nervous system, in particular the nervous system cytoskeleton, may contribute here. Indeed, it seems to make some sense. Reduction in gravitational force leads to a reduction in the prestress of the actin network within neurons, which leads to depression and indeed space flight reduces the staining of actin fibres in human monocytes [49], perhaps similarly to neurons? Indeed, it is of interest that there seems to be few examples of astronauts suffering schizophrenia-spectrum problems during or following missions.

## 9. The Situation in Plants

Far less work has been done on tensegrity and the cytoskeleton in plants than in animals, let alone with gravity factored in. Plants are certainly affected in microgravity [50] with reduced yield. However, in some species that can withstand hurricanes, cellulose microfibrils that make up the walls of the cable-fibres are oriented parallel to the long axis of the fibres and make the cables strong under tension. Dorsal and ventral leaf surfaces and partitions contain lignified fibre bundles and vascular strands that are strong under compression [51]. Possibly importantly, the microtubule cytoskeleton is intimately involved in the deposition of cellulose in the cell wall.

A model envisaging the cytoplasm as pervaded by an actin-based cytoskeletal network that is denser in the ER-devoid central region than in the ER-rich cell cortex and is linked to stretch receptors in the plasma membrane has been proposed for plants. Sedimenting statoliths may produce a directional signal by locally disrupting the network, thus, altering the balance of forces acting on receptors in different plasma membrane regions [52]. This may turn out to be vitally important if we are to explore space further. Plants provide food and produce valuable oxygen gas.

## 10. Concluding Remarks

As, in the future, longer and more ambitious extra-terrestrial missions are planned, the health of astronauts in microgravity will be paramount. I have demonstrated a possible role for the nervous system cytoskeletal tensegrity network in the mental health of astronauts. Life has arisen through eons on a planet with a considerable gravitational field [53]. It would be strange if there were not consequences for departing from it for considerable lengths of time [28]. Interestingly, it has been observed that astronauts grow in outer space by a solid inch or two, and their bodies deteriorate. This syndrome has similarities to the genetic disease acromegaly. While acromegaly may not directly involve the cytoskeleton, it is possible that cellular tensegrity is affected at least in the skin [54].

Are physical and mental changes experienced by astronauts pathogenic or adaptive? [55] They may assist existence in space but be crippling upon a return to a region of relatively high gravity. I am reminded of the navigators in Frank Herbert’s Dune Sci-Fi series who morph into something other in order to guide spaceships to the stars. If it will take generations to travel so far, there will be children conceived, born, and matured in microgravity [56]. What will the effect of microgravity on cytoskeletal proteins mean for them? Brave new world.

## Data Availability

Not applicable.
